# Experimental and theoretical analyses of nano-silver for antibacterial activity based on differential crystal growth temperatures

**DOI:** 10.1016/j.sjbs.2021.09.058

**Published:** 2021-09-27

**Authors:** Tariq Munir, Arslan Mahmood, Fahad Shafiq, Muhammad Fakhar-e-Alam, Muhammad Atif, Ali Raza, Shafiq Ahmad, Khurram Saleem Alimgeer, Nadeem Abbas

**Affiliations:** a Department of Physics, Government College University Faisalabad (GCUF), Allama Iqbal, Road, Faisalabad 38000, Pakistan; b Institute of Molecular Biology and Biotechnology (IMBB), The University of Lahore, 54590, Pakistan; c Department of Physics and Astronomy, College of Science, King Saud University Riyadh 11451, Saudi Arabia; d Industrial Engineering Department, College of Engineering, King Saud University, PO Box 800, Riyadh 11421, Saudi Arabia; e Electrical and Computer Engineering Department, COMSATS University Islamabad, Islamabad Campus, Pakistan; f Departments of Chemistry, University of Leicester, University Road, Leicester LE1 7RH, UK

**Keywords:** Antibacterial assay, *E. coli*, Nano-silver, Solution evaporation method

## Abstract

The modulation of antimicrobial properties of nanomaterials can be achieved through various physical and chemical processes, which ultimately affect subsequent properties. In this study, the antibacterial potential of nano-silver was investigated at 0.5, 1.0, 2.0, and 3.0 g/L, and its differential temperature synthesis was achieved at 20, 50, and 70 °C using the solvent evaporation method. Nano-silver particles exhibited FCC (octahedral) crystalline structure with crystallite sizes ranging between 28 and 39 nm calculated using XRD analysis. Moreover, irregular and non-uniform surface morphology was evident from SEM micrographs. The UV–Vis absorbance spectrum of nano-silver exhibited wave maxima at 433 nm, while the FTIR analysis depicted different modes of vibration indicating the CH, OH, C≡C, C-Cl, and CH_2_ functional groups attached to the surface. Lastly, nano-silver caused prominent inhibition (12.5 mm) in the *Escherichia coli* growth, particularly at 70 °C synthesis temperature and 3.0 g/L dose. It is concluded that both the nano-silver crystal growth temperature and dose contributed substantially to bacterial growth inhibition linked with subsequent size, shape-dependent properties.

## Highlights

• Nano-silver synthesized via solution evaporation method with octahedral crystal structure and crystallite size of 28 to 39 nm.
• Irregular and non-uniform surface morphology.
• Different functional on the surface of nano-silver included CH, CH_2_, OH, alkyne, and an alkyl halide.
• Nano-silver synthesized at 70 °C and a dose of 3.0 g/L caused effective inhibition of *E. coli.*

## Graphical abstract

**Figure:**
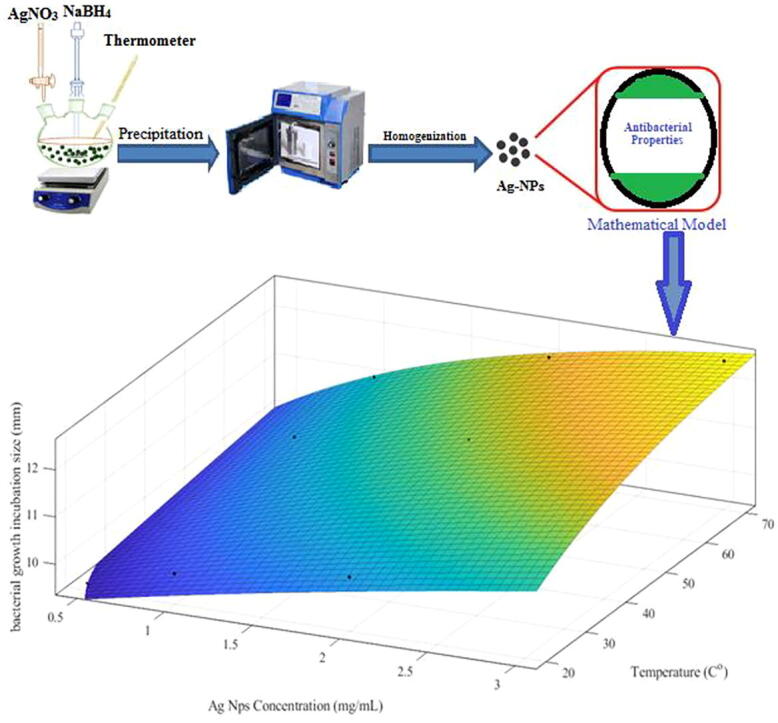

## 1 Introduction

Nanoparticle synthesis can be achieved via chemical and biological approaches. The bottom-up techniques recombine atoms or molecules into NPs while the top-down approaches convert bulk material into NPs. On a comparative basis, chemical synthesis methods (Bottom-up) are relatively cheaper and require less energy than top-down physical methods (Gabrielyan et al., 2019, Munir et al., 2019, Naskar et al., 2020, Erci et al., 2020, Danbature et al., 2021, Atif et al., 2021, Iqbal et al., 2021). It is essential to mention that various metallic oxides synthesized via bottom-up chemical approaches are potential candidates as antibiotics, and one prominent candidate is nano-silver (Ag-NPs).

The Ag-NPs exhibit multiple oxidation states such as (Ag^o^, Ag^2+,^ and Ag^3+^), leading to profound antibacterial effects. These are already used in various products of commercial and household importance (Gajbhiye and Sakharwade, 2016, Gunawan et al., 2017, Khan et al., 2018). In addition, the smaller diameter contributes to improved cellular penetration and can cause membrane damage and lipid peroxidation leading to bacterial cell death (Bondarenko et al., 2018). It is also reported that exposure to silver nanoparticles can lead to enhanced generation of reactive oxygen species that trigger bacterial cell death and promote subsequent antibacterial properties (Su et al., 2009, Song et al., 2019). Moreover, nano-silver could form electron-deficient Ag^+^ species that react with thiol groups of different cellular proteins in bacteria leading to protein denaturation (Liu et al., 2006, Mukha et al., 2013). The binding and interaction of Ag^+^ with bacterial enzymes disrupt the mitochondrial electron flow, thereby causing oxidative stress due to leakage of high-energy electrons (Holt and Bard, 2005, Bruna et al., 2021). Therefore, the antibacterial potential of nano-silver is substantial and can be utilized in the context of nano-medicine.

Microbes are an essential part of the environment and can cause various diseases in humans and other living organisms. One such bacterium is *Escherichia coli,* and it can cause food poisoning, diarrhea, and even pneumonia (Hati et al., 2018, La Combe et al., 2019) and 90 % of urinary tract infections in humans (Manges, 2016). Previous reports suggested antibacterial properties of the nanosilver against *E. coli* (Chalova et al., 2009, Touchon et al., 2009). Here for the first time, we present experimental and theoretical analyses of nano-silver synthesized via solution evaporation method using a two-way approach (both synthesis temperature and dose–response).

## 2 Experimental procedures

### 2.1 Synthesis of nano-silver

Sodium borohydride (0.02 M) solution was prepared in de-ionized water (200 mL) and incubated in an ice bath for 3 h. Afterward, the silver nitrate (0.01 M) was prepared in 10 mL de-ionized water with continuous stirring for 5 min. Next, the silver nitrate solution was added drop by drop into sodium borohydride solution with continuous stirring on a magnetic stirrer for 40 min at 20, 50, and 70 °C temperatures yielding a black-colored product. Finally, the product was filtered using filter paper, oven-dried at 200 °C for 5 h, and finely homogenized into nano-silver using mortar and pestle.

### 2.2 Characterization of nano-silver

Multiple characterization techniques like XRD, SEM, FTIR, UV–Vis were utilized to analyze the nano-silver final product. The XRD with model number D8 Advance, Bruker) X'Pert3 MRD XL) Cu-Kα radiation 1.5406 Å was used to determine the phase and material composition. The crystallite size was calculated by using Debye-Scherrer’s formula (Equation i).

(1) $$D=\frac{k\lambda }{\beta cos\theta }$$

Furthermore, SEM Emcrafts tabletop was used to collect the information about surface morphology while FTIR (Spectrum 2, Perkin Elmer) provided the information about fingerprints. Finally, UV–VIS was utilized to study the absorbance characteristics (Lambda 2, Perkin Elmer, LP74 Processor Module).

### 2.3 Culturing of *E. Coli* and antibacterial assay

The culturing and anti-bacterial assay was performed by using agar well diffusion medium (Invitrogen). After solidifying the media, *E. coli* cotton swabs were used for culturing bacteria, followed by incubation at 37 °C for 18–24 h. Different concentrations of 0.5, 1.0, 2.0, and 3.0 g/ L of Ag-NPs were applied for the antibacterial assay, and bacterial growth inhibition zone changes were investigated.

### 2.4 Mathematical modeling

Exponential function on both horizontal and vertical axis is selected as candidate function for the mathematical model using Mat-lab. The function is proposed to fit the data with reasonable goodness of fit as provided in SSE, R- square, adjusted R-square, and RMSE, and graphical representation is provided accordingly. In addition, the bacterial growth was calculated by using the following equation (ii);

(2) $$Bacterial\;growth={a+(12-b\times e}^{m\times Temp})+{(12-c\times e}^{-d\times Nano-silverConc.})+{(12-e\times e}^{-f\times nano-silverConc.\times Temp})$$

a, b, c, d, e, f, and m are unknown coefficients extracted through the least square error method with a 95% confidence index (*a = –32.68; b = 0.003465; c = -8.932; d = -0.02035; e = 3.332; f = 0.01095; m = -0.6369*).

## 3 Results

### 3.1 Characterization of nano-silver

#### 3.1.1 X-ray diffraction analyses (XRD) of nano-silver

**Fig. 1:**
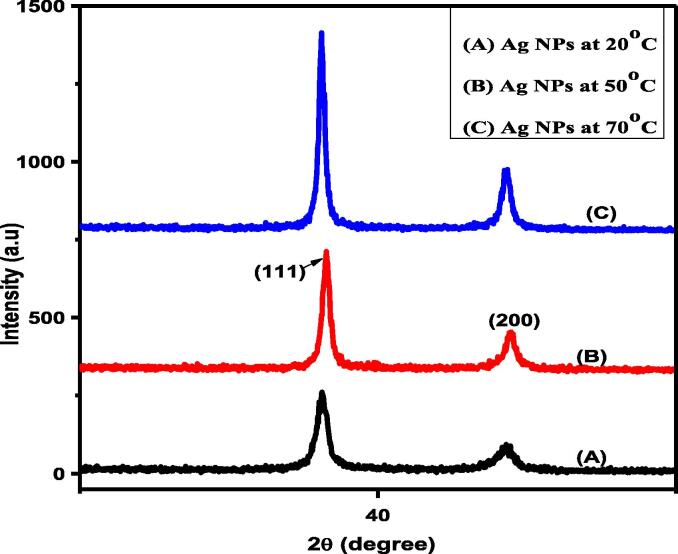
XRD spectrums of Ag-NPs synthesized at different temperature regimes.

The miller indices of different diffracted peaks were calculated (1 1 1) and (2 0 0) to confirm the crystalline nature of nano-silver. The miller indices of these peaks were compared with standard card JCPDS number 4–0783. The Bragg reflection 2θ at 38.12° and 44.35° and the FCC octahedral crystal structure was determined at (1 1 1). Moreover, the XRD spectrum of nano-silver synthesized at different temperature regimes depicted that an increase in the temperature increased the plasmonic band's intensity level while decreasing the nano-silver diameter (Fig. 1). The average crystallite size of Ag-NPs synthesized at varying temperature is given in Table 1.

**Table 1:** Average crystallite size of Ag-NPs synthesized at varying temperature.

Synthesis temperature of Ag NPs	Peaks (1 1 1)	Peaks (2 0 0)	Average crystallite size ranges (nm)
20 °C	29	28	28.5
50 °C	32	33	32.5
70 °C	38	40	39

#### 3.1.2 Scanning electron micrographs (SEM)

**Fig. 2:**
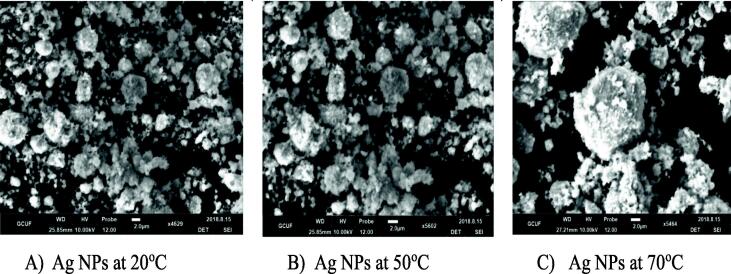
SEM analysis of Ag NPs synthesized at different temperature regimes.

The SEM micrographs of the surface morphology (Fig. 2A-C) and images were collected same scale range at 2 µm and represent the irregular and non-uniform surface of silver NPs at varying temperatures. An increase in the individual grain with increasing temperatures was evident due to the aggregation of larger particles which modified the surface morphology (spherical small and large) and grain size of nano-silver.

#### 3.1.3 Fourier-Transform infrared spectrum (FT-IR)

**Fig. 3:**
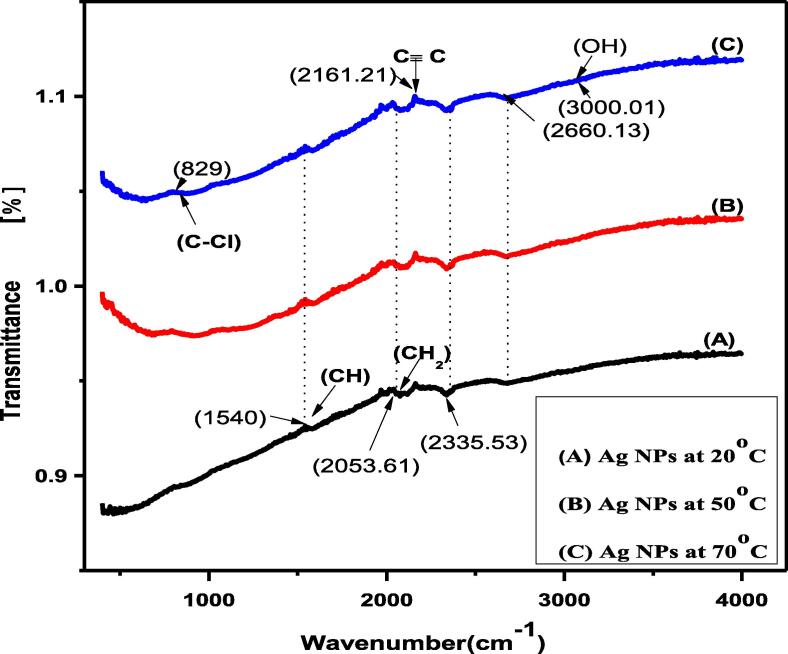
FTIR spectrum of Ag NPs synthesized at different temperature regimes.

The FTIR analysis revealed different functional groups attached to the surface of nano-silver (Fig. 3). The spectrum provided multiple modes such as CH, C-Cl, C≡C, CH_2,_ and OH. The strong absorbance was recorded at 1540 cm^−1^ and 2335.53 cm^-1^due to the vibration of alkyle group (C-H). Moreover, the modes at 2660 cm^−1^ also indicated slight vibration of alkyl group as compared to the previous 2335.53 cm^−1^ mode. The stretching at 2161.21 cm^−1^ indicated the alkyne (≡) group and the mode 829 cm^−1^ represents the alkyl halide (C-Cl).

#### 3.1.4 Ultraviolet–Visible absorption spectrum (UV–Vis)

**Fig. 4:**
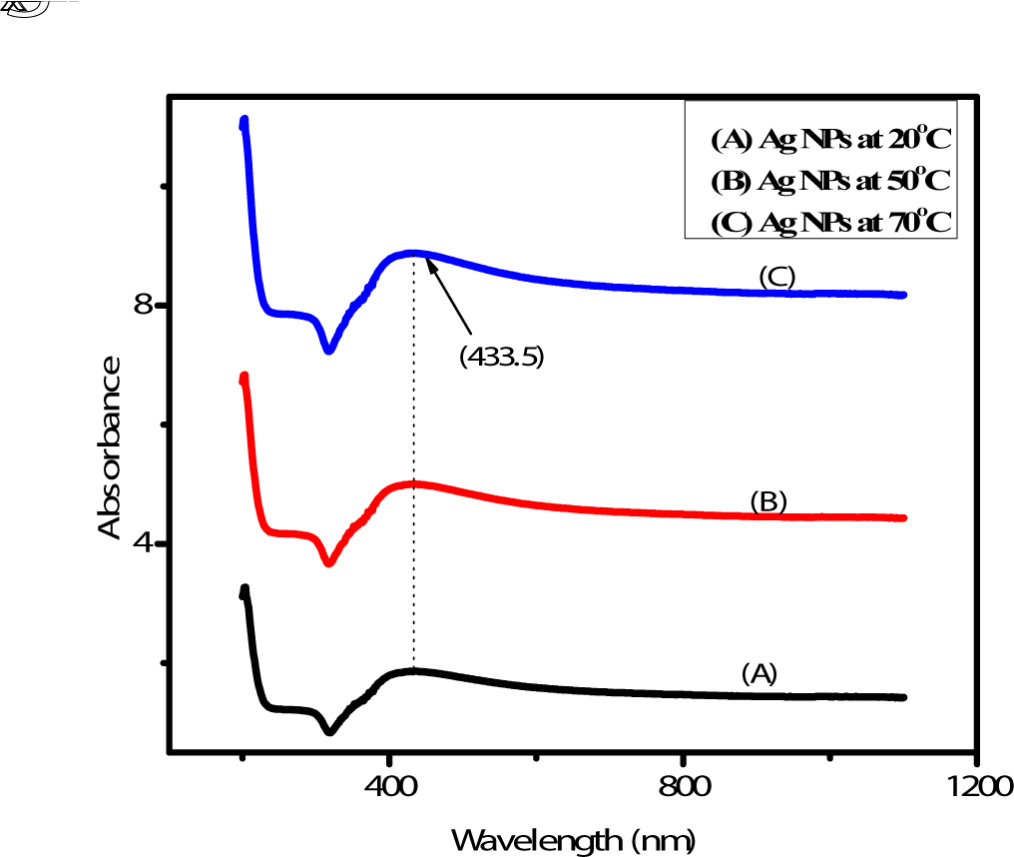
UV– visible absorbance spectra of the synthesized Ag-NPs.

The absorption characteristics of nano-silver are presented in Fig. 4. The absorption maxima at 433 nm corresponding to plasmon resonance were recorded, corresponding to the nano-silver optical behavior.

### 3.2 Antibacterial assay and the corresponding mathematical analyses

#### 3.2.1 Antibacterial assay

**Fig. 5:**
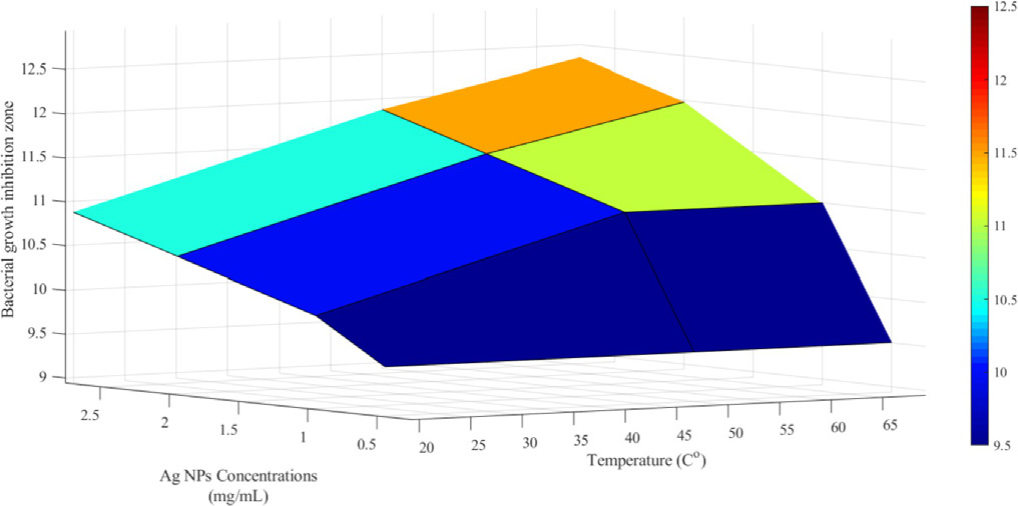
Bacterial growth incubation dependency on Ag NPs Concentration (g/L) and temperature.

Antibacterial effects of nano-silver at different concentrations (0.5, 1.0, 2.0 and 3.0 g/L) synthesized at different temperatures (20, 50 and 70 °C) varied significantly (Table 2). It was evident that bacterial growth was substantially inhibited at a higher dose (3.0 g/L) and 70 °C temperature of Ag-NPs, evident from the inhibition zone (Fig. 5). This also revealed that the nano-silver was more effective compared to other growth temperatures and doses.

**Table 2:** Inhibition zones and the concentration of Ag NPs synthesized at different temperatures.

Ag NPs concentrations (g/L)	Bacterial growth inhibition zone (size, mm)		
	20 °C	50 °C	70 °C
0.5	9.5	9.5	9.5
1	10	11	11
2	10.5	11.5	12
3	11.0	12	12.5

#### 3.2.2 Contour-plot and mathematical modelling

**Fig. 6:**
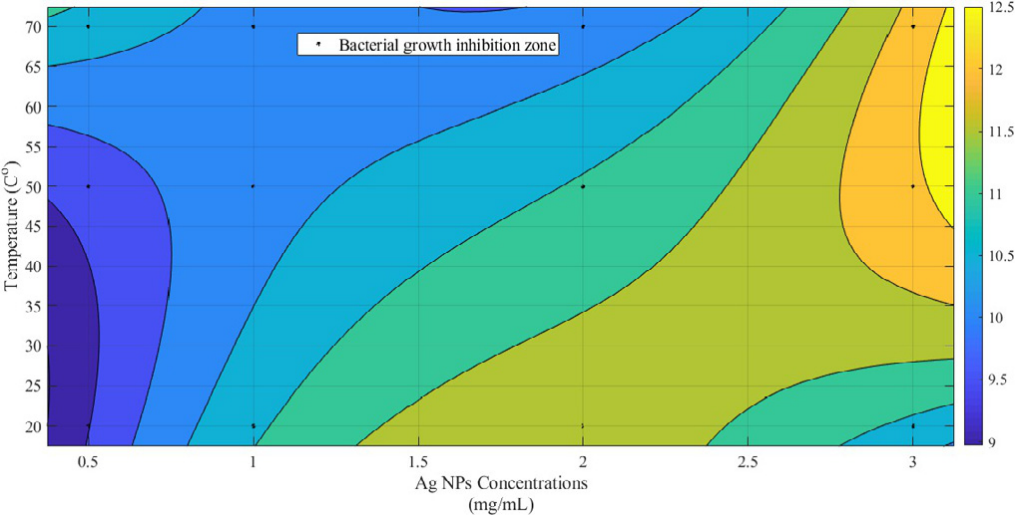
Contour plot of the bacterial growth with variation of Ag NPs concentration (mg/mL) and temperature (^o^C).

Likewise, the contour plot of the bacterial growth indicated dependency on the synthesis temperature and nano-silver dose (Fig. 6). It can be observed that the gradient of change is more with towards the right top corner, moving from the bottom left corner. This more significant change in gradient highlighted the effect of higher values of nano-silver dose and temperature. Conversely, lower changes at lower values of temperature and dose (g/L) exhibited lesser bacterial growth inhibition.

**Fig. 7:**
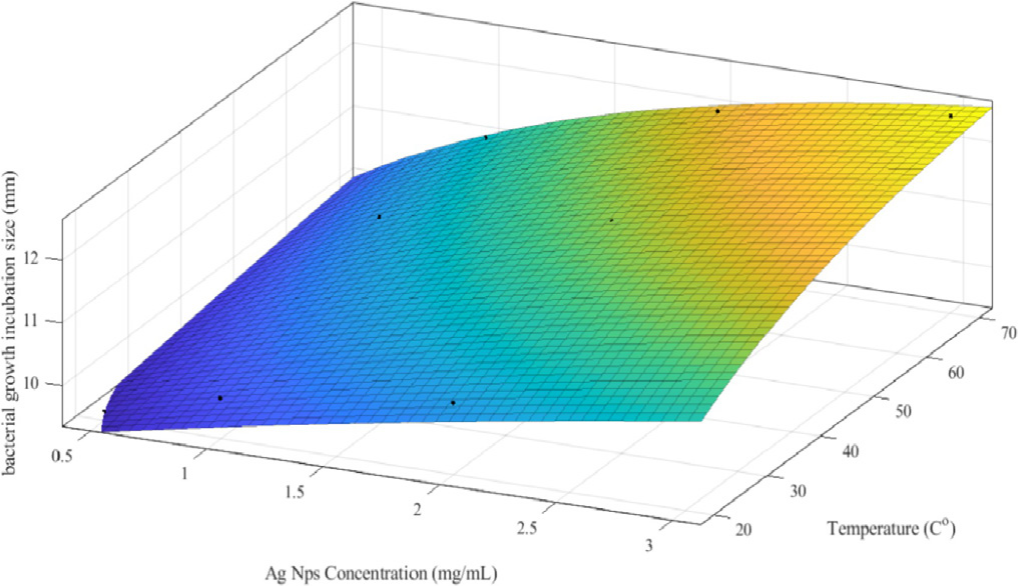
Experimental data comparison with the proposed mathematical model provided in equation (ii), along with the goodness of fit parameters and constants extracted through the method of least square errors.

The mathematical model was prepared using curve fitting and the method of least square errors. Exponential function on both horizontal and vertical axis is selected as candidate function for the mathematical model after analyzing the shape of the surface (Fig. 7). The following function is proposed to fit the data with reasonable goodness of fit, as evident from the figure of merits provided in SSE, R-square, adjusted R-square, and RMSE. It was also observed that goodness of fit is showing reasonable values to represent the data points. The SSE = 0.8026; R-square = 0.934; Adjusted R-square = 0.8549; RMSE = 0.4007. Lower values of SSE and RMSE reflected that the error representing the data with equation (ii) is low. Values close to 1 for R-square and adjusted R-square depicted a reasonable fit. The data points and the surface generated from the function are presented in equation (ii) and It is observed that experimental data and surface generated from equation (ii) are in good agreement (Fig. 7).

### 3.3 Discussion

Diffracted peaks were calculated at (1 1 1) and (2 0 0); miller indices of these peaks compared with standard card JCPDS number 4–0783 (Lanje et al., 2010). The octahedral crystal structure and crystallite size of pure nano-silver were increased by increasing the temperature and is consistent with the previous work (Singh et al., 2010, Allafchian et al., 2016, Munir et al., 2021). It is also reported that an increase in the temperature increased the intensity level of the plasmonic band and decreased the nano-silver diameter (Dada et al., 2018). Temperature-dependent increase in aggregation of larger particles and nano-silver grain modification agrees with the previously published work (Shahjahan et al., 2017, Munir et al., 2021). Besides, the 3000.01 (OH) and (CH_2_) 2053.61 cm^−1^ are responsible for reducing AgNO_3_ to Ag-NPs The given functional groups are attached on the surface of nano-silver as reported by (Muzamil et al., 2014, Hamedi et al., 2017, Elumalai et al., 2017). The UV–Vis absorption pattern also reflected nano-silver particle size increase with increasing temperature (Ninganagouda et al., 2014, Mekkawy et al., 2017).

We recorded prominent antibacterial activity and a different response based on synthesis temperatures. Increasing temperature above 70 °C correlated with the particles size and increased bacterial death linked with higher cellular NPs penetration (Gandhi and Khan, 2016, Tang and Zheng, 2018). Another possible reason for the better antibacterial action of Ag-NPs is variable oxidation states like Ag^o^, Ag^2+,^ and Ag^3+,^ which can influence bacterial cell death. The contour plot highlighted the effect of higher nano-silver dose and temperature values, and lower changes at lower values of temperature and dose (g/L) exhibited lesser bacterial growth inhibition. Earlier studies have also reported that the non-hazardous low-cost synthesis of silver NPs is also known as bactericidal nanomaterials (Slavin et al., 2017).

The present study reported the antibacterial effect of silver NPs against *E. coli* with the help of mathematical modeling. The Mat-lab used to operate the different exponential function to calculate the following parameter such as SSE = 0.8026; R^2^ = 0.934; RMSE = 0.4007. The R-square approximately equal to 1 indicated that fitness is reasonably well. Here, compare the dose of nano-silver with temperature, the higher dose of nano-silver increased bacteria death and vice versa. Lastly, the temperature-dependent changes at lower doses of nano-silver were non-significant, while increasing the dose of nano-silver mediated significant effects of crystal growth temperatures on bacterial inhibition.

## 4 Conclusions

The octahedral crystal structure and crystallite size of 28 to 39 nm, and irregular morphology of nano-silver were still effective in promoting inhibition of bacterial growth. Different functional on the surface of nano-silver included CH, CH_2_, OH, alkyne, and an alkyl halide, and it showed *λ_max_* at 433 nm. Moreover, an antibacterial assay performed against *E*. *coli* indicated that nano-silver synthesized at 70 °C and 3.0 g/L concentration resulted in an effective inhibition zone (12.5 mm) confirmed by the mathematical modeling approach. It is concluded that the nano-silver can be an effective antimicrobial agent, and it will be interesting to investigate the bioactive potential of nano-silver against different bacterial pathogens resistant to conventional drugs

## Declaration of Competing Interest

The authors declare that they have no known competing financial interests or personal relationships that could have appeared to influence the work reported in this paper.
